# Rationale and design of EXPLORE: a randomized, prospective, multicenter trial investigating the impact of recanalization of a chronic total occlusion on left ventricular function in patients after primary percutaneous coronary intervention for acute ST-elevation myocardial infarction

**DOI:** 10.1186/1745-6215-11-89

**Published:** 2010-09-21

**Authors:** René J van der Schaaf, Bimmer E Claessen, Loes P Hoebers, Niels J Verouden, Jacques J Koolen, Maarten J Suttorp, Emanuele Barbato, Matthijs Bax, Bradley H Strauss, Göran K Olivecrona, Vegard Tuseth, Dietmar Glogar, Truls Råmunddal, Jan G Tijssen, Jan J Piek, José PS Henriques

**Affiliations:** 1 Department of Cardiology, Academic Medical Center - University of Amsterdam, Amsterdam, the Netherlands; 2 Department of Cardiology, Onze Lieve Vrouwe Gasthuis, Amsterdam, the Netherlands; 3 Department of Cardiology, Catharina Ziekenhuis, Eindhoven, the Netherlands; 4 Department of Cardiology, Sint Antonius Ziekenhuis, Nieuwegein, the Netherlands; 5 Department of Cardiology, Onze Lieve Vrouwe Ziekenhuis, Aalst, Belgium; 6 Department of Cardiology, Haga Teaching Hospital, The Hague, the Netherlands; 7 Department of Cardiology, Sunnybrook Health Sciences Centre, Toronto, Canada; 8 Department of Cardiology, Lund University Hospital, Lund, Sweden; 9 Department of Cardiology, Helse Bergen Hospital, Bergen, Norway; 10 Department of Cardiology, Medical University of Vienna, Vienna, Austria; 11 Department of Cardiology, Sahlgrenska University Hospital, Gothenburg, Sweden

## Abstract

**Background:**

In the setting of primary percutaneous coronary intervention, patients with a chronic total occlusion in a non-infarct related artery were recently identified as a high-risk subgroup. It is unclear whether ST-elevation myocardial infarction patients with a chronic total occlusion in a non-infarct related artery should undergo additional percutaneous coronary intervention of the chronic total occlusion on top of optimal medical therapy shortly after primary percutaneous coronary intervention. Possible beneficial effects include reduction in adverse left ventricular remodeling and preservation of global left ventricular function and improved clinical outcome during future coronary events.

**Methods/Design:**

The Evaluating Xience V and left ventricular function in Percutaneous coronary intervention on occLusiOns afteR ST-Elevation myocardial infarction (EXPLORE) trial is a randomized, prospective, multicenter, two-arm trial with blinded evaluation of endpoints. Three hundred patients after primary percutaneous coronary intervention for ST-elevation myocardial infarction with a chronic total occlusion in a non-infarct related artery are randomized to either elective percutaneous coronary intervention of the chronic total occlusion within seven days or standard medical treatment. When assigned to the invasive arm, an everolimus-eluting coronary stent is used. Primary endpoints are left ventricular ejection fraction and left ventricular end-diastolic volume assessed by cardiac Magnetic Resonance Imaging at four months. Clinical follow-up will continue until five years.

**Discussion:**

The ongoing EXPLORE trial is the first randomized clinical trial powered to investigate whether recanalization of a chronic total occlusion in a non-infarct related artery after primary percutaneous coronary intervention for ST-elevation myocardial infarction results in a better preserved residual left ventricular ejection fraction, reduced end-diastolic volume and enhanced clinical outcome.

**Trial registration:**

trialregister.nl NTR1108.

## Background

Treatment of patients with acute ST-elevation myocardial infarction (STEMI) aims at early restoration of antegrade flow in the infarct related coronary artery in order to preserve myocardial function and improve survival. Angiography after thrombolysis or before primary percutaneous coronary intervention (PCI) has revealed that multivessel disease (MVD) is present in 40-65% of all STEMI patients. These patients are considered to be a subgroup with a high risk for morbidity and mortality, compared with patients with single vessel disease (SVD).[1,2] An aggressive multivessel PCI strategy during and after primary PCI for STEMI has not improved outcome in MVD patients. In fact, studies reported that treatment of non-culprit lesions in STEMI patients with MVD is associated with a higher post-procedural morbidity rate without improving survival.[3-5] In the setting of primary PCI it was recently demonstrated that, the higher mortality in patients with MVD is mainly determined by the presence of a chronic total occlusion (CTO) in a non-infarct related artery (IRA).[6-10] Furthermore, a CTO in a non-IRA was associated with a reduced left ventricular function during hospitalization for the index event and a further reduction in left ventricular function during follow-up.[6] Therefore, STEMI patients with a CTO constitute the MVD patient group with a truly higher risk for death.[6-9] The ongoing Evaluating Xience V and left ventricular function in PCI on occLusiOns afteR STEMI (EXPLORE) trial is the first randomized clinical trial powered to investigate clinical outcome after percutaneous treatment of a CTO. There are two main mechanisms involved in the hypothesis of the Explore trial. First, recanalization of the CTO will possibly restore the contractile function of the hibernating myocardium. Furthermore, recanalization of the CTO might improve the healing of the infarct border zone. This assumption is based on the coronary anatomy where the perfusion area of the infarct related coronary artery and the CTO are adjacent or overlapping. In recently perfused myocardium, the revascularization of a CTO will improve the myocardial perfusion in this overlapping region and therefore might improve the healing of this border zone and might protect against negative remodelling and preserving residual left ventricular function.

The EXPLORE trial will determine whether recanalization of a CTO within one week after primary PCI for STEMI results in a better preserved residual left ventricular ejection fraction and reduced end-diastolic volume.

## Methods/Design

### Overview

The investigator-initiated EXPLORE trial is a prospective, randomized, two-arm trial with blinded evaluation of endpoints. European and North-American high volume primary PCI centers with a 1.5 Tesla cardiovascular magnetic resonance imaging (cMRI) facility participate in this global trial. The study was first approved by the Medical Ethics Committee on human research at the Academic Medical Center-University of Amsterdam, the Netherlands. For all the foreign centers, the study must be approved by the Medical Ethics Committee on human research in each of the corresponding countries before participation. The study will be conducted in accordance with the declaration of Helsinki. The EXPLORE trial was registered on 30-10-2007 at http://www.trialregister.nl with trial ID NTR1108.[11]

**Figure 1:**
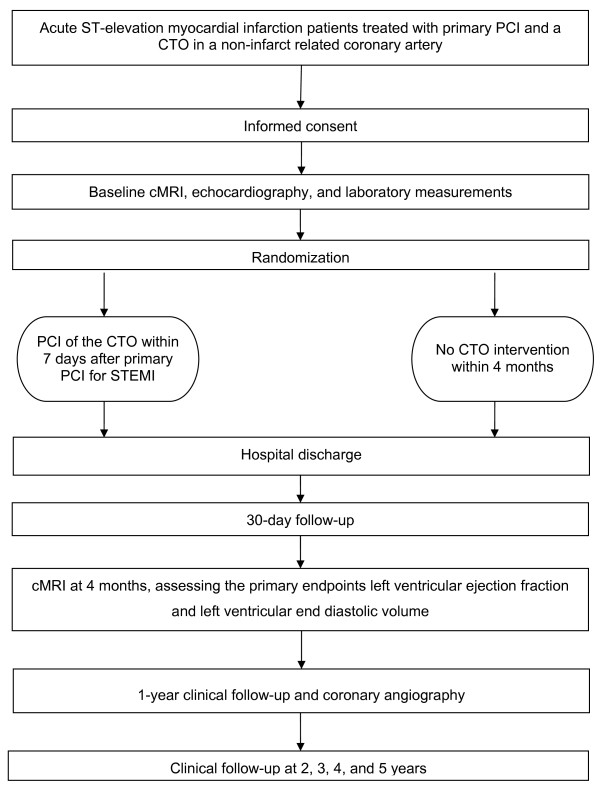
**Flow chart of the EXPLORE trial**. STEMI = ST elevation myocardial infarction PCI = Percutaneous Coronary Intervention CTO = Chronic Total Occlusion

After successful primary PCI for STEMI, patients with a CTO in a non-infarct related artery are randomized to either elective PCI of the CTO within seven days after primary PCI or to standard medical treatment. Figure 1 shows the flow chart of the trial.

### Patients and Enrolment

Consecutive STEMI patients after successful primary PCI, defined as a residual stenosis of the culprit lesion < 30% and a TIMI flow ≥ 2, are screened for entry into the EXPLORE trial. Patients are suitable for inclusion if coronary angiography preceding the primary PCI reveals at least one CTO, situated in a non-infarct related coronary artery or its side branches. For the purpose of this trial, a CTO is defined as a 100% luminal narrowing without antegrade flow or with antegrade or retrograde filling through collateral vessels. Furthermore, the CTO should be amenable to percutaneous treatment and must be located in a coronary vessel with a reference diameter of at least 2.5 millimeters. All inclusion and exclusion criteria are summarized in Appendix A and B, respectively.

If all inclusion and none of the exclusion criteria are met, the patient is asked for written informed consent, as required by the institutional review board in accordance with the Declaration of Helsinki.

### Randomized Treatment Assignment

After informed consent has been obtained, the local investigator contacts the EXPLORE website http://www.explorerandomization.org for online randomization. Patients are randomly assigned following a computer-generated list in a 1:1 ratio to either PCI of the CTO within seven days after the primary PCI or to standard medical treatment.

### PCI of the CTO(s)

All PCI procedures are performed under the local routine protocols. All angiograms are recorded in such a way that they are suitable for off-line quantitative coronary angiography (QCA). For patients randomized to PCI of the CTO, the procedure is planned as soon as possible after randomization, but at the latest within seven days after the primary PCI. An approved drug eluting stent is used during PCI of the CTO. For the purpose of uniformity in this trial, the investigators intend to use an everolimus eluting stent (EES) in order to study the performance of the EES in this type of lesion with a high risk of restenosis. Routine electrocardiography and blood analysis including cardiac enzymes are performed before and after PCI of the CTO and are repeated at discharge. Patients randomized to this treatment arm receive clopidogrel 75 mg daily or prasugrel 10 mg daily for at least 12 months after drug eluting stent placement [12,13].

### Standard Medical Treatment

In patients randomized to standard medical treatment, the non-infarct related CTO should not be approached invasively during the first four months after randomization. If, however an unequivocal indication for revascularization arises, follow-up cMRI should be performed before CTO intervention.

### Post-STEMI Treatment for Patients with non CTO co-existing coronary lesions

In view of the weak guideline recommendations [14] and the different local practices, our protocol recommends a conservative approach for non-CTO co-existing lesions in the EXPLORE trial. In order to assess the value of CTO revascularization but in view of current daily clinical practice, the decision of the management of non-CTO co-existing lesions must be made before randomization. This decision is made by the local heart team, which generally consist of an interventional cardiologist and or a cardio-thoracic surgeon. In case it is decided that a non-CTO co-existing lesion should be treated, this additional PCI procedure must be performed within one week after the index STEMI. For patients randomized to recanalize the CTO, this additional PCI procedure will be performed during the same procedure as the CTO lesion is being treated. For patients in the conservative group, this additional PCI for non-CTO co-existing lesions will be scheduled within one week after the index STEMI. After the first week, revascularization is only indicated when clinically and or ischemia driven and is preferably performed after four months.

### Post-STEMI Treatment for all Patients

All patients included in this trial are treated according to the current ACC/AHA guidelines regarding post STEMI management specifying treatment with at least 100 mg of aspirin daily, clopidogrel in a dosage of at least 75 mg daily or prasugrel 10 mg daily for at least 12 month after primary PCI, adequate lipid-lowering medication, angiotensin converting enzyme (ACE) inhibitors, and beta-blockers.[12,13]

### Follow-up

All patients visit the outpatient clinic at 30 days, four months, and twelve months after primary PCI. Thereafter, patients will be followed-up by means of a telephone call at two, three, four, and five years. During all three follow-up outpatient clinic visits, clinical evaluation with a 12-lead electrocardiogram and an inventory of adverse events is obtained. Adverse events included in this inventory are cardiac and non-cardiac death, stent thrombosis according to the definition of the Academic Research Consortium (ARC)[15] myocardial re-infarction, all cardiac surgery, implantation of an implantable cardioverter-defibrillator device, clinically overt heart failure, repeat coronary angiography and repeat PCI, hospital admission for angina, stroke, and severe bleeding events.

At four months, cMRI is performed in all patients along with routine laboratory measurements and exercise testing. If a contraindication for cMRI has risen because of altered patient characteristics after inclusion, the protocol permits single positron emission computed tomography (SPECT) as a secondary and echocardiography as a tertiary modality for endpoint assessment.

Scheduling of repeat angiography is at the discretion of the treating physician. Repeat angiography is preferably performed at one year after randomization in all patients.

### Cardiac Magnetic Resonance Imaging

After written informed consent, baseline cMRI is recommended to be performed at least 48 hours after the primary PCI but before the patient is randomly assigned to one of the treatment arms. All patients undergo cMRI at four-month follow-up to assess the primary endpoints of this trial. Patients are studied on a clinical 1.5-Tesla scanner using a 4-element phased array cardiac receiver coil. For functional imaging, electrocardiogram-gated cine steady-state, free-precession magnetic resonance images are obtained during repeated breath holds in the 3 standard long-axis views (4 -, 3- and 2-chamber view). Additional short-axis slices are acquired, covering the entire left ventricle from base to apex, to examine regional and global left ventricular function. Late contrast-enhanced (LCE) images are acquired 10 minutes after administration of a gadolinium-based contrast agent with an inversion recovery, gradient-echo pulse sequence to identify the location and extent of myocardial infarction. The data are obtained with slice locations identical to the functional images. All MRI images are sent to an independent core laboratory for quality control and blinded central analysis. On the short axis cine slices, the endocardial and epicardial borders are outlined manually in end-diastolic and end-systolic images, excluding trabeculae and papillary muscles. Assessment of global left ventricular function is obtained by calculating left ventricular volumes, mass, and ejection fraction using the summation of slice method multiplied by slice distance.

### Primary Endpoints

The two primary endpoints of this trial are differences between the two treatment arms in left ventricular ejection fraction (LVEF) and left ventricular end-diastolic volume (LVEDV), assessed by cMRI at four months after primary PCI for STEMI, Appendix C.

### Safety and Other Secondary Endpoints

Safety will be determined by the assessment of Major Adverse Coronary Events (MACE) defined as cardiac death, peri-procedural myocardial infarction, myocardial infarction or any repeat coronary intervention at 30 days, 4 months and 1, 2, 3, 4, 5 years. Furthermore, safety is also determined by the occurrence of stent thrombosis. All safety endpoints are defined in accordance with the ARC [15]. The trial will be monitored by a Data and Safety Monitoring Board (DSMB) and the trial will be discontinued in case of safety concerns.

All other secondary endpoints are displayed in Appendix C.

A pre-specified subgroup analysis will be performed in which the primary outcome, Major Adverse Coronary Events (MACE) and suitable other secondary endpoints will be stratified according to the presence of non-CTO co-existing lesions.

### Statistical Considerations

Both primary and all secondary endpoints are displayed in Appendix C.

As this study has two primary endpoints, the Hochberg extension of the Bonferroni method for multiple comparisons will be used to test for statistical significance with an overall type I error rate less than or equal to 0.05 [16]. The statistical comparisons of the treatment arms with respect to the primary and secondary endpoints are performed using the independent-samples T-test, or Fisher's exact probability test in case of binary endpoints. All p-values are 2-sided. For clinical outcomes such as the incidence of major adverse cardiac events, Kaplan-Meier curves displaying the pattern of events over the four-month and one-year follow-up period are constructed. Statistical significance and 95-% confidence intervals are calculated using Cox' proportional hazards model.

The trial is powered to detect differences between the two groups in cMRI assessed LVEF and LVEDV at four months after STEMI. With 2 × 150 randomized patients, this trial has an 80% power to detect absolute differences of 4% in LVEF and 15 mL in LVEDV in favor of PCI of the CTO with a one-sided alpha of 5%. For this calculation we have assumed that PCI of a CTO is successful in 80% of cases. The mean global ejection fraction in patients with an untreated CTO is assumed to be 36% against 40% in patients with successful CTO treatment with a common standard deviation of 12%. Consequently, the expected global ejection fraction is 40% (0.8 × 41% + 0.2 × 36%) in patients randomized to CTO treatment against 36% in patients randomized to standard medical treatment. The calculation for the second primary endpoint is based on the assumption of a net mean LVEDV of 185 ml for patients randomized to CTO treatment and of 200 ml for patients randomized to standard medical treatment. The standard deviation for LVEDV was assumed to be 45 ml. Patients who have deceased before primary endpoint measurement will be attributed the lowest LVEF and the largest LVEDV measured in the whole study cohort. The primary endpoint will be analyzed on an intention-to-treat basis.

### Study Organization and Monitoring

An executive committee will supervise the EXPLORE trial, while a study coordination committee will coordinate the trial and perform QCA analysis. The steering committee is responsible for design and conduct of the study. An independent data and safety monitoring board watches over the ethics of conducting the study in accordance with the Declaration of Helsinki, monitors the patient safety, and reviews safety issues as the study progresses. All major adverse cardiac events will be adjudicated by a Critical Events Committee. The specific role and information regarding each of the committees appear in appendix D.

## Discussion

### Prognosis after primary PCI

After primary PCI for acute STEMI, patients with MVD have a worse clinical outcome when compared to patients with SVD.[1,2,6,17] Previous randomized studies investigating multivessel PCI during the primary procedure or shortly thereafter have been hampered by small patient numbers and have failed to show clinical benefit of additional revascularization after primary PCI.[18,19] Furthermore, a number of observational studies investigating the value of additional revascularization after primary PCI have reported inconclusive results.[3-5,20-22]

Recently, we reported that patients with a CTO in a non-IRA are the subgroup of MVD patients who are truly at risk after primary PCI for STEMI.[6,8] A CTO in a non-IRA was a strong and independent predictor of 30-day, 1-year, and 5-year mortality whereas MVD without a CTO was only a weak predictor of 30-day mortality, and not an independent predictor of 1-year, and 5-year mortality. We reported similar results in a cohort consisting exclusively of STEMI patients with cardiogenic shock.[9] Furthermore, a CTO in a non-IRA was associated with reduced LVEF during the index hospitalization and a further reduction in LVEF within the first year thereafter.[6] Therefore, rather than a significant diseased concomitant coronary artery, a CTO in a non-IRA is a target for additional revascularization after primary PCI.

These findings drive the rationale behind the EXPLORE trial. Recanalization of a CTO in a non-IRA shortly after primary PCI may improve regional myocardial function and promote infarct healing at the border zones. These effects may attenuate the remodeling process, which may lead to a better preserved residual global LV function, decreased LVEDV, and improved survival. This trial will be the first to prospectively determine the effect of PCI of a CTO in a non-IRA in the early recovery phase after successful primary PCI for STEMI on LV performance.

### LV function after elective PCI of a CTO

LVEF and LVEDV are major prognostic determinants in patients with coronary artery disease. It is suggested that opening of CTO's in an elective setting can be of benefit by restoring blood flow to hibernating myocardium and thus improving LV function. Improvement of LV function and a reduction of both end-diastolic and end-systolic volume after recanalization of a CTO has been demonstrated in several studies, provided long-term patency could be achieved.[23-26]

### PCI of a CTO: higher risk of restenosis

Percutaneous recanalization of a CTO amenable to PCI treatment can be performed with a success rate of 70-80%, but is associated with a higher rate of restenosis compared to PCI of non-occluded vessels. Although coronary stenting has been shown to be superior to conventional balloon angioplasty, restenosis rates remain relatively high. When compared to bare metal stents, drug eluting stents (DES) are effective in decreasing the need for repeat intervention in successfully treated CTO patients.[27] However, there have been concerns about long-term delay of arterial healing as a consequence of both Sirolimus eluting stent (SES) and Paclitaxel eluting stent (PES) placement and the associated risk of late stent thrombosis. Both preclinical and clinical data for a second-generation DES, the EES, are encouraging in terms of arterial healing and low restenosis rates. The EES showed superior endothelialization compared with the PES, SES and Zotarolimus-eluting stent at 14 days after stent implantation.[28] This suggests a superior safety profile for the EES, as shown in the one-year results of the SPIRIT IV trial, randomizing 3687 patients in a 2:1 fashion to either the EES or the PES for treatment of patients with de novo coronary artery disease with a maximum of 3 lesions. The SPIRIT IV investigators reported a 0.29% ARC definite or probable stent thrombosis rate in the EES arm, as compared to 1.1% in the PES arm (p = 0.003). Furthermore, the EES performed superior over the PES regarding ischemia-driven target lesion revascularization, (3.9% vs 6.6%, p = 0.0008).[29] Although extensively studied in relatively low-risk lesions, currently data regarding the performance of the EES in CTO's are lacking.

To conclude, no clinical trial to date has investigated the effect of percutaneous recanalization of CTO's on clinical endpoints in any setting. The high case-fatality rates and inferior recovery of LV function after STEMI of patients with a CTO in a non-IRA provide the rationale for the current trial. The ongoing EXPLORE trial is the first randomized clinical trial powered to investigate clinical outcome after percutaneous treatment of a CTO. The Explore trial will determine whether recanalization of a CTO within one week after primary PCI for STEMI results in a better preserved residual left ventricular ejection fraction and reduced end-diastolic volume.

## Appendix A

### Inclusion criteria EXPLORE trial

Patients are eligible for inclusion if all of the inclusion criteria are met:

- Successful primary percutaneous coronary intervention^1 ^for acute ST elevation myocardial infarction^2^

And

- Presence of at least one chronic total occlusion located in a non-infarct related coronary artery, defined as a 100% luminal narrowing without antegrade flow or with antegrade or retrograde filling through collateral vessels

And

- Reference diameter of ≥2.5 millimeters

And

- Amenable to treatment by percutaneous coronary intervention

1 Residual stenosis of the culprit lesion < 30% and Thrombolysis In Myocardial Infarction flow ≥ 2.

2 definition according to Alpert et al. Myocardial infarction redefined - a consensus document of The joint ESC/ACC committee for the redefinition of myocardial infarction [30].

## Appendix B

### Exclusion criteria EXPLORE trial

Age > 80 years

Persistent or permanent atrial fibrillation

Known renal insufficiency (serum creatinin > 265 μmol/L or > 3.5 mg/L)

Persistent hemodynamic instability^1 ^lasting up to 48 hours after primary PCI

Cardiac events^2 ^between primary PCI and randomization

Significant left main stenosis (diameter stenosis ≥ 50%)

Severe coronary artery disease, not amenable for PCI but suitable for coronary artery bypass grafting

Severe valvular heart disease requiring cardiac surgery within four months

Clinically driven indication for implantable cardioverter defibrillator within four months

Inability to schedule the index procedure within seven days after primary PCI

Contraindication for cMRI (i.e. pacemaker, cerebrovascular clips, or claustrophobia)

Serious known concomitant disease with a life expectancy of less than one year

Circumstances that prevent follow-up

Previous participation in this trial or any other trial within the previous 30 days

1 Defined as pre-shock (heart rate > 100/min, and/or systolic blood pressure < 100 mm Hg) or shock

2 Defined as extended myocardial infarction, acute stent thrombosis, or late (> 48 hours after primary PCI) and life threatening ventricular arrythmias

PCI = Percutanous Coronary Intervention, cMRI = cardiovascular Magnetic Resonance Imaging

## Appendix C

### EXPLORE trial endpoints

#### Primary Endpoints

Measured by cardiovascular magnetic resonance imaging at 4 months:

- Left ventricular ejection fraction

- Left ventricular end-diastolic volume

### Secondary Endpoints

#### Safety Endpoints

Major Adverse Cardiac Events, defined as cardiac death, myocardial infarction or any repeat coronary

intervention* at 30 days, 4 months, and 1, 2, 3, 4, and 5 years

Stent thrombosis, classified as definite, probable or possible

#### Other Secondary Endpoints

Measured by cardiac magnetic resonance imaging at 4 months:

- Left ventricular end-systolic volume - Left ventricular segmental wall thickening

- Left ventricular mass - Infarct size (by late contrast enhancement)

N Terminal - pro Brain Natriuretic Peptide (at 4 months and 1 year, relative to baseline)

Heart rate-adjusted QT (QTc) duration measured by resting electrocardiography (at 4 months and 1 year, relative to baseline)

Quantitative Coronary Angiography of the treated chronic total occlusions:

- in-stent and in-segment late luminal loss at 12 months

- in-stent and in-segment minimal luminal diameter

- in-stent and in-segment binary restenosis rate

Repeat Hospitalization for cardiac causes at 30 days, 4 months, and 1, 2, 3, 4, and 5 years

Presence of clinically overt heart failure at 30 days, 4 months, and 1, 2, 3, 4, and 5 years

Implantation of implantable cardioverter-defibrillator devices

Functional Class: NYHA classification at 30 days, 4 months, and 1, 2, 3, 4, and 5 years

*Excluding the initial percutaneous coronary intervention of the chronic total occlusion when randomly

assigned to this treatment arm. MRI: Magnetic resonance imaging NYHA: New York Heart Association

## Appendix D

### Executive Committee

The Executive Committee will be composed of the study principal investigators and selected members among the investigators. This committee is responsible for overseeing the administrative progress of the study and approval of the final trial design and protocol issued to the DSMB and the clinical sites. This committee will also be responsible for reviewing the final results, determining the methods of presentation and publication, and selecting the secondary projects and publications by members of the Steering Committee. The executive committee also holds the right to modify or stop the study prematurely based on recommendations from the DSMB.

### Steering Committee

The Steering Committee will be composed of the principal investigators from the participating centers in this trial. The Steering Committee is responsible for the day-to-day administrative management of the trial and will meet on a monthly basis to monitor subject enrollment, clinical site progress and protocol compliance. It will be the responsibility of the Steering Committee to provide assistance and education to individual sites and researchers to help with trial management, record keeping, and reporting requirements.

### Data Safety Monitoring Board

The Data Safety Monitoring Board (DSMB) will be composed of general and interventional cardiologists and a biostatistician. The DSMB will function in accordance with applicable regulatory guidelines. The board members are independent and will not be participating in the trial. The DSMB will review the safety data from this trial and will make recommendations based on safety analyses of unanticipated device effects, serious adverse events, protocol deviation, and device failures. The DSMB will meet annually; in addition, the DSMB may meet at any time if there is reason to suspect that safety is an issue. The DSMB is responsible for making recommendations regarding any safety or compliance issues throughout the course of the study and may recommend to the Executive Committee to modify or stop the study. However, all final decisions regarding study modifications rest with the Executive Committee. All cumulative safety data will be reported to the DSMB and reviewed on an ongoing basis throughout enrollment and follow-up periods to ensure patient safety. Every effort will be made to allow the DSMB to conduct an unbiased review of patient safety information. The DSMB will develop a consensus understanding of all trial endpoints and definitions used in the event adjudication process. All DSMB reports will remain strictly confidential but will be made available to the regulatory body upon request.

### Critical Events Committee

The Critical Events Committee (CEC) consists of two experienced clinical cardiologist who will not participate in the trial. The CEC will adjudicate all major adverse cardiac events in this trial as specified by the study protocol. Both members of the CEC will be blinded to the primary results of the trial.

## Appendix E

### Participating Centers and Representatives

José PS Henriques, Academic Medical Center, University of Amsterdam, Amsterdam, the Netherlands

René J van der Schaaf, Onze Lieve Vrouwe Gasthuis, Amsterdam, the Netherlands

Jacques J Koolen, Catharina Ziekenhuis Eindhoven, Eindhoven, the Netherlands

Maarten J Suttorp, Sint Antonius Ziekenhuis, Nieuwegein, the Netherlands

Matthijs Bax, Haga Ziekenhuizen, The Hague, the Netherlands

Emmanuele Barbato, Onze Lieve Vrouwe Ziekenhuis, Aalst, Belgium

Göran K. Olivercrona, Lund University Medical Center, Lund, Sweden

Truls Råmunddal, Sahlgrenska University Hospital, Gothenburg, Sweden

Vegard Tuseth, Helse Bergen Hospital, Bergen, Norway

Dietmar Glogar, Medical University of Vienna, Vienna, Austria

Bradley H. Strauss, Sunnybrook Health sciences centre, Toronto, Canada

## Competing interests

As principal investigator, J.H. has received a research grant to fund and coordinate this project. All other authors confirm that they have no competing interests to declare.

## Authors' contributions

RS: participated in the conception and design of the study, wrote and refined the manuscript and has given approval of the final version. BC: participated in the design and coordination of the study, wrote and refined the manuscript. LH: participated in the design and coordination of the study, wrote and refined the manuscript. NV: participated in the conception and design of the study and revised the manuscript. JK, MS, EB, MB, BS, GO, VT, DG, TR: revised the manuscript. JT: participated in the conception and design of the study, developed the statistical analysis plan and revised the manuscript. JP: participated in the conception and design of the study and revised the manuscript. JH: participated in the conception and design of the study, refined and revised the manuscript, and has given his approval of the final version.
